# Comparative adsorptive behaviour of cow dung ash and starch as potential eco-friendly matrices for controlled organophosphorus pesticides delivery

**DOI:** 10.1038/s41598-022-15292-6

**Published:** 2022-07-01

**Authors:** Chinyere Emmanuella Okafor, Ikenna Onyido

**Affiliations:** 1 Department of Science Education, Chukwuemeka Odumegwu Ojukwu University, Uli, Anambra State Nigeria; 2 Department of Pure and Industrial Chemistry, Nnamdi Azikiwe University, Awka, Nigeria

**Keywords:** Environmental sciences, Environmental impact, Chemical safety, Environmental chemistry, Materials chemistry, Surface chemistry

## Abstract

The work reported herein explores the viability of cow dung ash (CDA) as a matrix for controlled pesticide delivery, by comparing its adsorptive characteristics towards two organophosphorus pesticides with those of starch, conventionally utilized in designing controlled pesticide delivery systems. CDA was characterized by Fourier transform infrared (FTIR) spectroscopy and powder X-ray diffraction (PXRD). Data for pesticide adsorption on the surfaces correlate well with Langmuir and Freundlich isotherms, with the former isotherm giving a slightly better fit (*R*^2^ ≥ 0.90) than the latter (*R*^2^ ≥ 0.81). Values of the adsorption parameters *K*_*L*_ and *R*_*L*_ indicate favourable pesticide adsorption on both surfaces. Desorption is the microscopic reverse of adsorption; both processes obey pseudo-second-order kinetics. The implication of this kinetic form is a mechanism in which adsorbate diffusion to the polymer surface and its transport into the polymer interior are important events. The isothermal and kinetic ratios, $\frac{{K_{L}^{CDA} }}{{K_{L}^{Starch} }} = 3.8$ and 4.0, $\frac{{k_{2}^{CDA} }}{{k_{2}^{Starch} }} = 1.3$ and 0.6, and $\frac{{k_{ - 2}^{CDA} }}{{k_{ - 2}^{Starch} }} = 5.2$ and 1.0 at pH 7.0 and 27 °C, compare the adsorptive behaviour of diazinon and dichlorvos, respectively, on CDA and starch. These parameters are of the same order of magnitude, signalling that CDA is as potentially viable as starch for use as a matrix for pesticide-controlled delivery.

## Introduction

Intensification of agricultural production in the drive towards food security as envisioned in the Sustainable Development Goals (SDGs)^1^ nvolves expansion of farmlands and increased use of farm inputs, such as fertilizers and pesticides. The use of pesticides is associated with environmental pollution^2^, loss of biodiversity^3^, damage to human health^4,5^, etc. Sustainable resource management requires the adoption of mitigation strategies that reduce environmental and human health risks associated with increased use of pesticides. This has led to the search for approaches which deliver pesticides directly to targets, thereby minimizing the dissipation of the chemicals in the environment^6^ and the resulting ecotoxicity. A strategy which has caught our interest is the encapsulation of pesticides in eco-friendly matrices to obtain controlled release formulations (CRFs). This shields the pesticides from direct interactions with humans and the environment during their delivery and in the course of their pesticidal action^7^. First utilized in medicine to regulate drug delivery rates in patients^8,9^, this strategy is now a familiar approach in modern medicine^10^.

The matrices used for preparing controlled release formulations in this Age of Sustainable Development^11^ must be naturally occurring, biodegradable and biocompatible to scale the sustainability test^12^. Ideally, such materials should also be unfit for human consumption. This is to prevent the quest for environmental sustainability from undermining food security by encroaching on the aggregate food supply and agro-based industrial feedstocks, as happens in the competition between biofuels and food security^13^. Such matrices should also be inexpensive so that, ultimately, low-cost technologies derived from their use can be accessed by farmers, including resource-poor farmers who abound in developing countries^14^. Biodegradable matrices so far encountered in controlled pesticide delivery systems are biopolymers such as the polysaccharides starch, cellulose, lignin, chitosan, dextran, agarose, alginates, (sometimes) proteinaceous materials such as gelatin and albumin^15^, in addition to kaolin^16,17^ and kaolinite^18^ surfaces in a number of occasions.

Cow dung is a faecal waste freely available in rural farming communities, in animal markets and in formal and artisanal abattoirs. The bulk of available cow dung is largely regarded as waste in many communities. Cow dung has found use as soil amendments^19^, as heating and cooking fuel^20^, in biogas production^21^, in mud-brick manufacture for housing^22^, in compost manure^23^, and in reducing bacterial and pathogenic diseases in traditional medicine^24,25^. Some of these uses pose collateral threats to the health of users. To the best of our knowledge, cow dung has not been explored as a matrix for controlled pesticide release formulations. Its biodegradability, biocompatibility, and ready availability at little or no financial costs make it an ideal material for the development of low-cost technologies for increased food production in the context of environmental sustainability. Such low-cost agricultural technologies would ensure that the interests of resource poor farmers, who dominate the agricultural production space in developing countries, are well protected. The focus of this paper is to seek an understanding of the basic adsorptive characteristics of cow dung, in the form of cow dung ash (CDA), towards commonly utilized pesticides, before studies on its capabilities as a controlled release matrix are embarked upon.

We report in this paper an exploratory study of the adsorption on and desorption from cow dung ash (see below) of two commonly used organophosphorus pesticides in developing countries, diazinon (**1**) and dichlorvors (**2**). Diazinon is a moderately hazardous Class II pesticide^26^, while dichlorvos is extremely toxic to non-target organisms^27^. The results obtained are compared with data also obtained in this study under the same experimental conditions for starch, a more conventional matrix, in order to preview the prospects of using CDA as a substitute for starch as a controlled delivery matrix for the two pesticides.
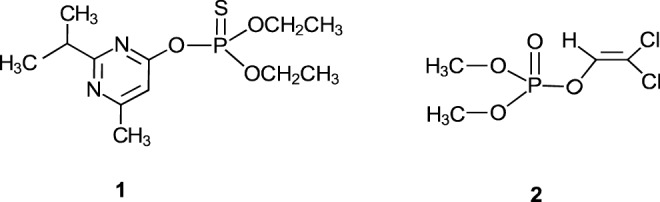


## Materials and methods

### Materials

The following chemicals were utilized in this study in the manners indicated. Diazinon, an organophosphorus pesticide whose adsorptive characteristics were investigated in this study, potassium hydrogen phthalate and potassium dihydrogen phosphate used to control the pH of the media utilized in the adsorption studies, and ninhydrin used for the derivatization of dichlorvos, were all Sigma-Aldrich products. Dichlorvos, the second pesticide investigated and sodium tetraborate decahydrate used to control medium pH were all technical grade products from Merck. Corn starch used as an adsorbent in the study as well as sodium hydroxide and hydrochloric acid used for standardization were all analytical grade chemicals obtained from the British Drug House, UK. These chemicals were used as supplied. The compositional concentrations of these chemicals are supplied in Table S1 in the Supplementary Information File. Cow dung samples were collected from Kwata cattle market, Uli, situated at latitude 5.78° N and longitude 6.82° E in Anambra State of Nigeria.

The pH of solutions was measured with a Meterlab PHM290 pH Stat Controller. Other instruments are described in the relevant sections of Results and Discussion below.

### Processing of cow dung to yield cow dung ash (CDA) for experimental use

The cow dung sample utilized in this study was wet at the point of collection. It was dried at room temperature (27 °C), ground in a mortar, and sieved to a particle size of ≤ 53 μm with a BSS 300 standard sieve. The dry, sieved sample of cow dung was first weighed in a crucible, then ashed in a furnace at 550 °C for an hour to obtain cow dung ash (CDA). The crucible with its CDA content was placed in a desiccator to cool to room temperature and was then weighed again. The difference in weight represented an 8.5% loss in weight on ignition of the cow dung sample to yield CDA. The resulting ash coloured CDA was stored in an airtight brown bottle protected from light and was subsequently used as required.

### Characterization of CDA

#### Fourier transform infrared spectroscopic analysis of CDA

Fourier transform infrared (FTIR) spectra of pulverized samples of CDA were obtained using a ThermoFisher Nicolet 3801 FT-IR operating in the range of 400–4000 cm^−1^ at a spectral resolution of 2 cm^−1^. A background scan of KBr was acquired before the CDA sample was scanned. The sample of CDA was blended with KBr and pelletized before measurement. 25 scans were accumulated within the spectral range and at the spectral resolution given above. The AIST Spectral Database for Organic Compounds facilitated FTIR peak assignments, in addition to other literature sources cited.

#### X-Ray diffraction patterns of CDA

CDA samples were first sieved onto the surface of a silicon disc pre-coated with petroleum jelly and then scanned on a ThermoFisher INEL Equinox 1000 X-ray diffractometer with a Cu radiation source from 0° to 140° (2*θ*).

### The adsorption–desorption equilibria of the pesticides on CDA and starch matrices

Adsorption equilibrium studies involving the pesticides on CDA and starch matrices were studied by the batch equilibrium method^28,29^. Aliquots of 10 ml buffer solution (pH = 4.0, 7.0 or 9.0) and 40 ml of a solution of the pesticide of known concentration were introduced into Teflon bottles each of which contained 250 mg of the adsorbent. The samples were vigorously agitated on a mechanical shaker at 250 rpm for 2 h at 27 °C. The resulting suspension was subsequently centrifuged at 4500 rpm for 10 min. Five ml portions of the supernatant in each bottle was withdrawn for spectrophotometric determination of the active ingredient (a.i). Each experiment was duplicated. Pesticide solutions in the buffer medium in the absence of the adsorbent were treated similarly to serve as blanks.

Desorption of the pesticides from CDA and starch surfaces was measured at pH 7.0 and 27 °C. Measurements were commenced immediately after adsorption equilibrium was attained. The adsorbent/adsorbate ratio was kept the same as in the adsorption measurements described above. Five ml of the supernatant was withdrawn for spectrophotometric analysis. This volume was replaced with 5 ml of the buffer solution in order to maintain the sink conditions.

### Kinetics of pesticide adsorption on CDA and starch

For each kinetics experiment, 250 mg of the adsorbent (CDA or starch) was weighed into capped bottles, followed by the addition of 10 ml buffer solution of pH 7.0 and 40 ml of the solution of the pesticide maintained at 27 °C. The capped bottles were placed on a mechanical shaker. At intervals of 0, 10, 20, 40, and 80 min, a vial was taken and centrifuged at 4500 rpm. Five ml of the supernatant solution was filtered through 0.2 μm syringe filters; its concentration was then determined spectrophotometrically.

### Kinetics of the desorption of the pesticides from CDA and starch surface into water

Desorption kinetics studies which were undertaken at pH 7.0 and 27 °C, commenced immediately after the kinetics of the adsorption. Ten ml of the buffer solution at pH 7.0 was poured into capped bottles containing the pesticide and the matrix. The bottles were shaken and then centrifuged. Five ml of the supernatant were withdrawn at known time intervals for spectrophotometric determination of the a.i. concentration.

### Spectrophotometric determination of concentrations

Solutions of diazinon have a well-defined *λ*_*max*_ at 264 nm, the wavelength used to obtain the molar photometric experimental readings. Solutions of dichlorvos, on the other hand, had no well-defined *λ*_*max*_ in the range of 200–800 nm. However, the reaction between the pesticide and ninhydrin gives the dichlorvos-ninhydrin complex which has a well-defined *λ*_*max*_ at 401 nm. Changes in the absorbance of the product solutions were related to their concentrations once the molar absorptivity, *ε*, was known. A modification of the method used by Tzaskos et al.^30^ for the derivatization of glyphosate with ninhydrin was used to estimate experimental concentrations of dichlorvos. A mixture of a known weight of dichlorvos and excess ninhydrin reagent prepared by the method of Moore^31^ was immersed in boiling water for 30 min and cooled in an ice-bath. After attaining room temperature, the resulting solution was diluted serially and their absorbances measured at 401 nm to obtain the calibration curve which enabled the calculation of *ε*.

## Results and discussion

### Molar absorptivity of diazinon and the ninhydrin derivative of dichlorvos

The molar absorptivity, *ε*, of diazinon and the ninhydrin derivative of dichlorvos, was measured as 1.73 × 10^4^ and 3.34 × 10^3^ L mol^−1^ cm^−1^, respectively, from the Beer–Lambert calibration plots given as Fig. S1 in the Supplementary Information. These molar absorptivity values enabled the conversion of experimental absorbances to pesticide concentrations by the application of the Beer-Lambert law.

### Fourier transform infrared (FTIR) spectra of CDA

Cow dung, when unprocessed, consists of *ca*. 80% water and undigested residues of fodder, faeces, urine, lignin, cellulose, hemicelluloses, amino acid residues from crude proteins, soil residues, an assortment of minerals, such as K, S, Fe, Mg, Ca, Co, Mn, etc.^32,33^. The relative proportion of these species in CDA would conceivably depend on the habitat in which the cattle is reared and how CDA was obtained from cow dung. Consequently, absorptions due to the O–H function in lignin and the celluloses, C–H stretches which abound in carbohydrate derivatives, the amide function and the N–H bond from metabolized proteins/amino acid residues, the Si–O bond stretching in SiO_2_ from soils, among others, would be expected in an IR spectrum of CDA.

The FTIR characteristics of starch are well documented in the literature. We have summarized the IR absorptions of starch from literature sources in Table 1. The FTIR absorptions of CDA from this work are also juxtaposed against those of starch in Table 1 to enable a comparison of the spectral properties of these two matrices.

**Table 1:** Band assignments^a,b^ for the FTIR spectra of CDA^a^ and starch^c^.

Band in the FTIR spectrum of CDA (cm^−1^)^a,b^	Assignment	Band in the FTIR spectrum of starch (cm^−1^)^b^	Assignment
3700–3000 centred at 3419	O–H stretching	3600–3000	O–H stretching
2918	CH_2_ deformation	3000–2800	CH_2_ deformation
2150	Combination of hindered rotation and OH bending	2100	Combination of hindered rotation and OH bending^d^
1638	C=O of amide residues	1642	O–H from adsorbed water
1420	Lignin CH_2_ deformation and cellulose CH_2_ stretch; alginate COO^−^ symmetric stretch^e^	1415	CH_2_ bending, C–O–O stretch
1157–1030	C–O–C vibrations of ether linkages and Si–O stretch	1242–1094	CH_2_OH, C–O, C–C stretching and C–O–H bending

^a^Data from this work. ^b^The AIST Spectral Database for Organic Compounds facilitated FTIR peak assignments, in addition to other literature sources cited. ^c^Data taken from ref. 38. ^d^See Ref. ^40^. ^e^Alginate was used in the preparation of CDA matrix (see caption to Fig. 1A).

**Figure 1:**
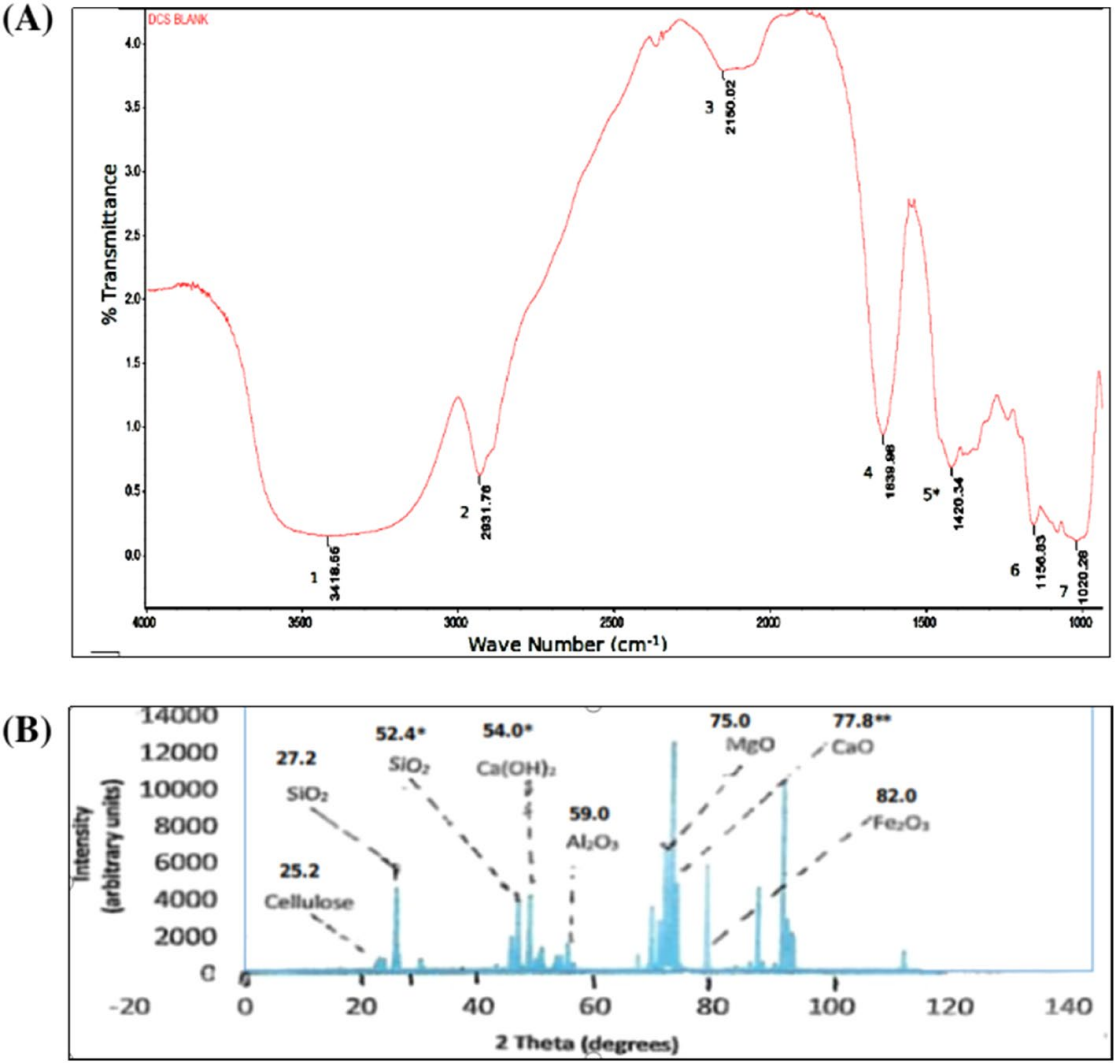
Spectroscopic characteristics of CDA: (**A**) Peak identification of FTIR spectrum of CDA used in this study as follows: 1 = 3419 cm^−1^, –OH; 2 = 2918 cm^−1^, –CH_2_–; 3 = 2150 cm^−1^, –OH; 4 = 1638 cm^−1^, C=O; 5 = 1420 cm^−1^, –CH_2_– and C–O–O; 6–7 = 1157–1030 cm^−1^, C–O–C, Si–O. *The sample was taken from CDA beads which contain alginate whose characteristic carboxylate –COO^−^ function also absorbs in the 1420 cm^−1^ region. (**B**) Peak identification of XRD pattern shown by CDA. *These peaks were assigned to these minerals in the CDA sample studied by Vishwakarma and Ramachandran (see Ref. ^45^). **The sample was taken from CDA beads which contain Ca^2+^ used for gelling the beads in our controlled release study (to be published) which is mainly responsible for the peak at 2*θ*° = 77.8.

Our attention is now focussed on the major absorptions in the 4000–1000 cm^−1^ region of the FTIR spectrum of CDA shown in Fig. 1A. The broad band between 3700 and 3000 cm^−1^, which is centred at 3419 cm^−1^, is due to the stretching vibration of the O–H group. This is consistent with the assignment by Ciolacu et al.^34^ and Kizil et al.^35^ for this functional group found in the celluloses, lignin, and starch. There is the possibility that this band for O–H vibration overlapped with the N–H asymmetric vibration in amino acid residues^36^. The band at 2918 cm^−1^ is assigned to the C–H vibration in cellulose and lignin^37^. The combined hindered rotation and O–H bending absorb at 2150 cm^−1^. The peak at 1638 cm^−1^ is attributed to the stretch of the carbonyl function in amide residues and in lignin^38^. The band at 1420 cm^−1^ is characteristic of the –CH_2_– deformation in lignin which is reinforced by the symmetric bending vibration of the same group in cellulose^34^. The symmetric vibration of the exogeneous alginate carboxylate also occurs in this region. The absorption bands in the region 1157–1030 cm^−1^ are assigned to the C–O–C vibrations of the diverse ether linkages present in the celluloses and to the Si–O stretch of soil in the CDA. The similarities in the FTIR absorptions of CDA and starch^34,35,39^ summarized in Table 1 as well as the similarities in the FTIR characteristics of CDA and those of cellulose/lignin^38,40,41^ may presage some similarities in the adsorption behaviour of these surfaces when considered from the structure–activity point of view.

### Powder X-ray diffraction (PXRD) spectrum of CDA

X-ray diffraction studies reveal the extent of crystallinity or otherwise^41,42^ of samples under investigation, in this case CDA. The X-ray diffractogram of a crystalline polymer sample yields sharp peaks while that of an amorphous sample gives diffuse peaks. The relatively sharp peaks obtained in the diffractogram in Fig. 1B gives the hint that the CDA utilized in this study is substantially crystalline. Since lignin is known to be largely amorphous and yields diffuse peaks^42,43^, it is reasonable to infer from Fig. 1B that the CDA matrix used in our study has significantly more amount of cellulose than lignin.

As noted earlier, CDA would contain an assortment of organic and mineral compounds whose proportion would depend on the habitat in which the cattle are reared and how the CDA was obtained from cow dung. For example, Avinash and Murugesan^42^, in a chemometric analysis of CDA, demonstrated that the following minerals showed XRD peaks at 2*θ*°: cellulose, 24.8; SiO_2_, 26.9; Al_2_O_3_, 61.1; MgO, 75.0; CaO, 78.4; Fe_2_O_3_, 80.5. These peaks are basically present, but shifted laterally in some cases, in the PXRD spectrum of CDA blank beads shown in Fig. 1B to give cellulose, 25.2; SiO_2_, 27.2; Al_2_O_3_, 59.0; MgO, 75.0; CaO, 77.8; and 82.0. We assign the same peaks to the same species, by analogy^42^. Vishwakarma and Ramachandran^44^, in their study of CDA-modified concrete assigned a peak at 2*θ*° = 52.4 to silica, as well as the peak at 2*θ*° = 54.0 to Ca(OH)_2_, in addition to the peaks listed and assigned above. These peaks assigned by Vishwakarma and Ramachandran^44^ are also present in the diffractogram in Fig. 1B.

Cellulose, from which lignin and hemicellulose have been removed, is known to exhibit enhanced crystalline character^43^. The absence of lignin and probably other organic materials in the spectrum in Fig. 1A may be attributed to the high temperature used to process cow dung, to obtain the CDA used in this work. Significant intermolecular hydrogen bonding in cellulose has also been shown to increase the crystallinity of cellulose^45^. The intensity of the peak at 2*θ*° = 78.4 probably reflects the presence of exogenic Ca which came from the gelling agent used in the formulation of the CDA beads. Overall, XRD diffractogram in Fig. 1B shows the presence of many minerals in the CDA sample used for this study which contributes substantially to its crystalline character.

### Adsorption–desorption equilibria of the pesticides on CDA and starch surfaces

The capacity of a matrix to adsorb a pesticide and how readily (or reluctantly) the surface releases the adsorbed species are important factors, among many, that determine the ability of the matrix to deliver pesticides to specific sites in controlled quantities. Adsorption–desorption data can provide useful information about the basic features of the adsorbing/desorbing system such as sorption mechanism, surface properties of the adsorbent and its affinity for the solute.

Adsorption equilibrium studies involving diazinon and dichlorvos on CDA and starch surface were undertaken by the batch equilibrium method^46,47^. The amount of the pesticide adsorbed, *q*_*e*_ (mg/g), was calculated on the basis of the principle of mass balance, according to Eq. (1), where *C*_0_ and *C*_*e*_ = initial and final (i.e., equilibrium) concentrations (mg/dm^3^), respectively, of the pesticide in the aqueous phase; *v* = volume of aqueous solution (dm^3^); and *w* = mass of adsorbent (g). The experimental data for the adsorption of diazinon and dichlorvos on CDA and starch at pH 4.0, 7.0 and 9.0 at 27° C are assembled in Tables S2–S5 (Supporting Information); these are now modelled after the Langmuir and Freundlich isotherms to ascertain which of these two isotherms gives a better fit with our data.

1 $$q_{e} = \left( {C_{0} - C_{e} } \right) \cdot \frac{v}{w}$$

### The Langmuir adsorption isotherm

The Langmuir isotherm^48^, applicable to homogeneous surfaces, is given by Eq. (2), where *K*_*L*_ = the maximum adsorption (mg/g) to form a monolayer of the a.i., *C*_*e*_ = equilibrium concentration of a.i. (mg/dm^3^) in the aqueous phase, *q*_*e*_ = amount of a.i. adsorbed per unit mass of adsorbent, *b* = Langmuir constant related to the affinity of the binding sites (mg/g) for sorbate molecules.

2 $$\frac{{C_{e} }}{{q_{e} }} = \frac{{C_{e} }}{{K_{L} }} + \frac{1}{{K_{L} b}}$$

Rearrangement of Eq. (2) yields Eq. (3); a plot of 1/*q*_*e*_* versus* 1/*C*_*e*_ should yield a straight line, from which the Langmuir constants *K*_*L*_ and *b* can be extracted. The essence of the Langmuir isotherm is also captured by a dimensionless separation factor, *R*_*L*_, defined by Eq. (4)^49,50^. The magnitude of *R*_*L*_ gives information about the favourability of the adsorption process or otherwise as follows: favourable if 0 < *R*_*L*_ < 1; unfavourable *R*_*L*_ > 1; linear if *R*_*L*_ = 1; and irreversible if *R*_*L*_ = 0.

3 $$\frac{1}{{q_{e} }} = \frac{1}{{K_{L} }} + \left( {\frac{1}{{K_{L} b}}} \right)\left( {\frac{1}{{C_{e} }}} \right)$$

4 $$R_{L} = \frac{1}{{1 + bC_{0} }}$$

**Figure 2:**
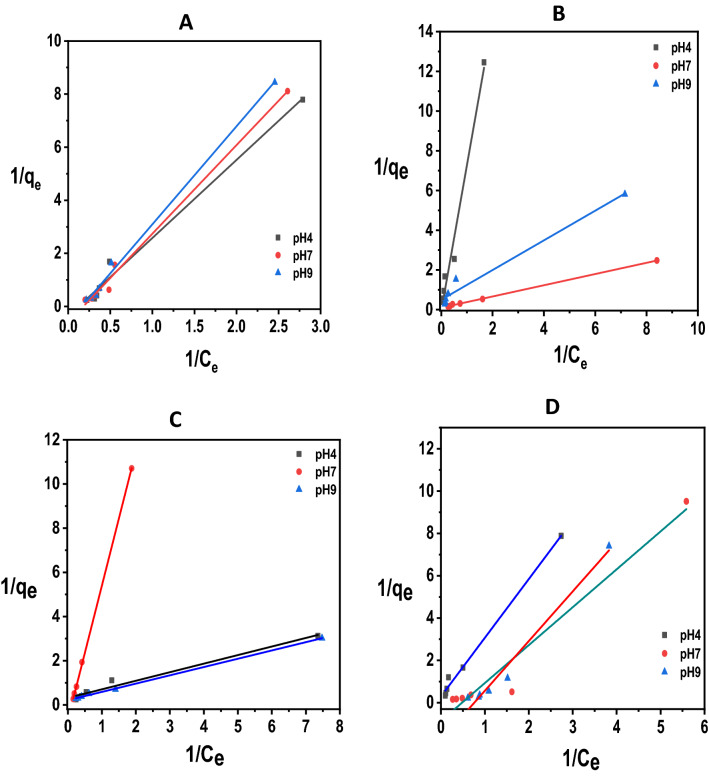
Langmuir isotherms at 27 °C and pHs 4.0, 7.0 and 9.0 for the adsorption of (**A**) diazinon and (**B**) dichlorvos on CDA and for the adsorption of (**C**) diazinon and (**D**) dichlorvos on starch.

Data for the adsorption of diazinon and dichlorvos on CDA and starch surfaces are collected in Tables S2–S5 (Supplementary Information). Plots of 1/*q*_*e*_* versus* 1/*C*_*e*_ at the three pHs studied for the adsorption of diazinon and dichlorvos on the two surfaces yield the Langmuir isotherms shown in Fig. 2, from which the Langmuir adsorption parameters assembled in Table 2 were extracted.

**Table 2:** Langmuir parameters (derived from the plots in Fig. 2) for the adsorption of diazinon and dichlorvos on CDA and starch surfaces at 27 °C and different pHs.

Matrix	Pesticide	pH	*K*_*L*_ (mg/g)	*b* (dm^3^/mg)	*R*_*L*_	*R*^2^ value	Δ*G*_*ads*_ (kJ mol^−1^)
CDA	Diazinon	4.0	2.76	0.12	0.31	0.988	− 2.5
		7.0	1.67	0.16	0.39	0.996	− 1.3
		9.0	1.67	0.18	0.33	0.993	− 1.3
	Dichlorvos	4.0	5.41	0.03	0.76	0.982	− 4.2
		7.0	2.22	0.33	0.19	0.982	− 2.0
		9.0	2.88	0.10	0.15	0.999	− 2.6
Starch	Diazinon	4.0	1.13	0.89	0.11	0.864	− 0.3
		7.0	0.44	0.64	0.08	0.922	+ 2.0
		9.0	0.69	0.68	0.11	0.927	+ 0.9
	Dichlorvos	4.0	3.40	0.11	0.43	0.993	− 3.1
		7.0	0.56	0.76	0.09	0.980	+ 1.4
		9.0	1.92	0.98	0.06	0.958	− 1.6

The linearity of the plots in Fig. 2 for which *R*^2^ values ≥ 0.9 (see Table 2) shows that the Langmuir isotherm is applicable to the system under study which, mechanistically, means that the surface of the adsorbent is covered by a monolayer of the adsorbate^51^. What is obvious from the data in Table 1 is that the adsorption capacity, *K*_*L*_, for both pesticides follows the pH order of 4.0 > 7.0 ≈ 9.0 on CDA surface, and the pH order of 4.0 > 9.0 > 7.0 on starch surface, which is to say that the adsorption of both adsorbates is more favourable in acidic solutions than in neutral and basic ones.

We suggest that this observed effect of pH on *K*_*L*_ is due to proton coordination with the basic sites on the adsorbents and adsorbates in the acidic medium which promotes hydrogen bonding and other non-covalent interactions^5^. This interaction, which is absent in neutral and basic media, enhances adsorption of the solutes on such surfaces. This idea is consistent with the increase of the surface charge of the adsorbent and the degree of ionization of the adsorbates which have been advanced to explain the influence of pH on adsorbing systems^50,52^. The ratio of the adsorptive capacity of the surface for dichlorvos, $K_{L}^{dichl}$, and for diazinon, $K_{L}^{diaz}$, i.e. $\frac{{K_{L}^{dichl} }}{{K_{L}^{diaz} }}$ is calculated as 2.0, 1.3, and 1.7 at pH 4.0, 7.0, and 9.0, respectively, for CDA surface and 3.0, 1.3, and 2.8 at pH 4.0, 7.0, and 9.0, respectively, for starch. The magnitude of this ratio shows that both surfaces have slightly higher capacities to adsorb dichlorvos than diazinon. The ratio of the surface adsorptive capacity of the two adsorbents, $\frac{{K_{L}^{CDA} }}{{K_{L}^{Starch} }}$, is also calculated as 2.4, 3.8, and 2.4 for diazinon and 1.6, 4.0, and 1.5 for dichlorvos at pH 4.0, 7.0, and 9.0, respectively. The magnitude of this ratio shows that CDA has slightly higher adsorptive capacities than starch for both pesticides at all the pHs investigated. It is to be noted, however, that these values of $\frac{{K_{L}^{CDA} }}{{K_{L}^{Starch} }}$ are of the same order of magnitude.

It has been argued^53,54^ that *K*_*L*_ does not represent a true thermodynamic function in adsorption processes. However chemical intuition suggests that *K*_*L*_ is impliedly related to the true thermodynamic equilibrium constant, $K_{eq}^{ads}$. In fact, Liu^55^ has shown that with uncharged solutes, *K*_*L*_ approximates to the true equilibrium constant, *K*_*ads*_. The solutes utilized in this study are organophosphorus esters which are uncharged in their standard states, for which the statement $K_{L} \approx K_{eq}^{ads}$ may be made, on the basis of Liu’s assertion. This enables the free energy change for adsorption, Δ*G*_*ads*_, to be obtained from the thermodynamic expression given in Eq. (5). The Δ*G*_*ads*_ values so obtained are included in Table 2. Admittedly, this method for obtaining the thermodynamic parameter, Δ*G*_*ads*_, suffers from the limitation that the components of Δ*G*_*ads*_, i.e. Δ*H*_*ads*_ and TΔ*S*_*ads*_, are not accessible through this same route.

5 $$\Delta G_{ads} = - RT\ln K_{L}$$

*R*_*L*_ values measured for the two adsorbates on the two surfaces at the different pHs are all < 1; this, from the definition of *R*_*L*_ outlined above^49,50^, is an indication that the adsorption of these species on the adsorbents is favourable, under the prevailing experimental conditions. The favourability of the adsorption process depicted by the magnitude of *R*_*L*_ is confirmed by the values of Δ*G*_*ads*_ obtained from the Langmuir *K*_*L*_ values, on the assumption above, that $K_{L} \approx K_{eq}^{ads}$, except for the cases of the adsorption of diazinon on starch at the pHs 7.0 and 9.0, as well as the adsorption of dichlorvos at pH 7.0, for which Δ*G*_*ads*_ is positive but small. The small but negative Δ*G*_*ads*_ values mostly observed accord with favourable adsorption of the physisorption type^56^.

### The Freundlich adsorption isotherm

The expression for the Freundlich isotherm^57^, applicable to heterogeneous surfaces, is given in Eq. (6), where *K*_*F*_ = the Freundlich adsorption capacity and *n* = adsorption intensity. If *n* > 1, the adsorption is deemed favourable^58^. The linear form of Eq. (6) is Eq. (7), from which it is seen that a plot of log *q*_*e*_ versus log *C*_*e*_ should give a straight line with slope = 1/*n* and intercept = log *K*_*F*_. The data for the adsorption of diazinon and dichlorvos on CDA and starch surfaces in Tables S2–S5 (Supplementary Information), respectively, are treated graphically as discussed above to obtain the Freundlich isotherms for the two pesticides on the surfaces. These plots are shown in Fig. S2 (Supplementary Information).

6 $$q_{e} = K_{F} C_{e}^{{{\raise0.7ex\hbox{$1$} \!\mathord{\left/ {\vphantom {1 n}}\right.\kern-\nulldelimiterspace} \!\lower0.7ex\hbox{$n$}}}}$$

7 $$\log q_{e} = \log K_{F} + \frac{1}{n}\log C_{e}$$

The Freundlich adsorption parameters resulting from these plots are collected in Table 3. As observed for the Langmuir adsorption isotherm above, the Freundlich adsorption capacity, *K*_*F*_, is higher for dichlorvos than diazinon. However, while *K*_*F*_ for dichlorvos is sensitive to medium pH as expected because it bears a site that could be protonated and follows the pH order of 4.0 > 7.0 ≈ 9.0, its value for diazinon is independent of pH. The largely positive values of this parameter indicate that adsorption by the Freundlich mechanism is also favourable^59^. Furthermore, the Freundlich isotherm pertains to adsorption on heterogenous surfaces with the capacity for multilayer adsorption of the adsorbate which allows for interaction between adsorbent molecules^60^. Significantly, Freundlich adsorption is of the chemisorption type in which chemical bonds hold the adsorbent and adsorbate molecules together^61^.

**Table 3:** Freundlich parameters (derived from the plots in Figs. S1 and S2 (Supplementary Information) for the adsorption of diazinon and dichlorvos on CDA and starch surfaces at 27 °C and different pHs.

Surface	Pesticide	pH	*K*_*F*_ (mg/g) (dm^3^/mg)^1/n^	*n*_*F*_ (dm^3^/mg)	*R*^2^ value
CDA	Diazinon	4.0	1.08	2.59	0.907
		7.0	1.08	2.38	0.959
		9.0	1.10	2.08	0.953
	Dichlorvos	4.0	5.02	3.60	0.926
		7.0	2.89	0.79	0.984
		9.0	2.98	5.61	0.854
Starch	Diazinon	4.0	1.18	3.82	0.843
		7.0	1.06	1.29	0.975
		9.0	1.03	1.00	0.941
	Dichlorvos	4.0	1.30	1.21	0.987
		7.0	1.10	1.86	0.808
		9.0	1.18	1.39	0.993

A close inspection of the adsorption parameters and *R*^2^ values gathered in Tables 2 and 3, which were derived from the plots displayed in Fig. S2 (Supplementary Information) and 2 shows that, although the experimental data for the adsorption of both adsorbates on the two matrices follow both the Langmuir and Freundlich models, slightly better fits are obtained with the Langmuir model (*R*^2^ ≥ 0.90), when compared to its Freundlich counterpart (*R*^2^ ≥ 0.81).

### Kinetics of the adsorption of diazinon and dichlorvos on CDA and starch surfaces

In this section, data for the adsorption of the adsorbates on CDA and starch, collected as a function of time and shown in Tables S6 and S7 (Supplementary Information) are modelled after the linear forms of zero-, first- and second-order rate equations outlined below, in order to probe the kinetic order which best describes the adsorption process on both surfaces.

If the concentration of the solute (i.e., adsorbate) in solution is *c* at time zero, and the amount of solute adsorbed onto the surface is *b* at time *t*, then the rate of adsorption for a zero-order process is given by Eq. (8)^62,63^, where *k*_*o*_ is the pseudo zero-order rate constant. According to Eq. (8), a plot of (*c* − *b*) against *t* should give a straight line with slope = *k*_0_. Such zero-order plots for the adsorption of diazinon and dichlorvos on CDA and starch surfaces are shown in Fig. S3 (Supplementary Information).

8 $$\left( {c - b} \right) = c - k_{0} t$$

9 $$\ln \left( {c - b} \right) = \ln c + k_{1} t$$

The rate equation for a first-order process is given by Eq. (9), where *k*_1_ is the pseudo first-order rate constant. According to this rate expression, a plot of ln (*c* − *b*) versus *t* should give a straight line with slope = *k*_1_. The kinetic plots for the adsorption of diazinon and dichlorvos on CDA and starch surfaces according to first-order behaviour are shown in Fig. S4 (Supplementary Information).

10 $$\frac{t}{{\left( {c - b} \right)}} = \frac{1}{{k_{2} }} \left( {c - b} \right)^{2} + \frac{t}{{\left( {c - b} \right)}}$$

**Figure 3:**
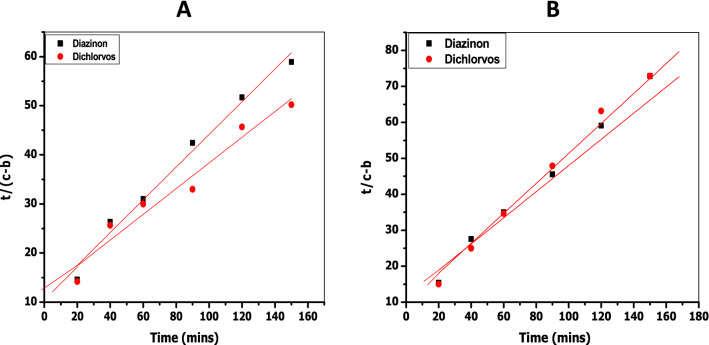
Second-order kinetic plots for (**A**) the adsorption of diazinon and dichlorvos on CDA surface and for (**B**) the adsorption of diazinon and dichlorvos on starch surface at pH 7.0 and 27 °C.

Equation (10) is the rate expression for a second-order process, where *k*_2_ is the pseudo second-order rate constant. A plot of $\frac{t}{{\left( {c - b} \right)}}$ against *t* according to Eq. (10) should give a straight line with slope = $\frac{1}{{\left( {c - b} \right)}}$ and intercept = $\frac{1}{{k_{2} }}\left( {c - b} \right)^{2}$. A combination of the slope and intercept would yield the pseudo second-order rate constant, *k*_2_. The kinetic plots for the adsorption of both adsorbates on CDA and starch according to second-order behaviour are shown in Fig. 3.

The rate constants and *R*^2^ values derived from the kinetic plots in Figs. S3, S4 (Supplementary Information) and 4 according to pseudo zero-, pseudo first-, and pseudo second-order adsorption behaviour, respectively, of the two pesticides on CDA and starch, are summarized in Table 4. It is seen that *R*^2^ values derived from the relevant plots are closest to unity for the second-order behaviour of dichlorvos adsorption. This suggests that second-order adsorption kinetics is applicable to this pesticide on both CDA and starch surfaces.

**Table 4:** Rate constants and *R*^2^ values obtained by modelling the adsorption of diazinon and dichlorvos on CDA and starch surfaces at pH 7.0 and 27 °C.

Surface	Pesticide	Order of reaction	Rate constant	*R*^2^ value
CDA	Diazinon	Zero-order	*k*_0_ = 9.1 × 10^−3^ mg min^−1^	0.962
		First-order	*k*_1_ = 4.7 × 10^−3^ min^−1^	0.928
		Second-order	*k*_2_ = 3.3 × 10^−1^ g mg^−1^ min^−1^	0.985
	Dichlorvos	Zero-order	*k*_0_ = 4.0 × 10^−2^ mg min^−1^	0.888
		First-order	*k*_1_ = 1.9 × 10^−3^ min^−1^	0.792
		Second-order	*k*_2_ = 2.6 × 10^−1^ g mg^−1^ min^−1^	0.970
Starch	Diazinon	Zero-order	*k*_0_ = 6.0 × 10^−3^ mg min^−1^	0.925
		First-order	*k*_1_ = 4.0 × 10^−3^ min^−1^	0.899
		Second-order	*k*_2_ = 4.6 × 10^−2^ g mg^−1^ min^−1^	0.987
	Dichlorvos	Zero-order	*k*_0_ = 5.0 × 10^−3^ mg min^−1^	0.888
		First-order	*k*_1_ = 3.0 × 10^−3^ min^−1^	0.855
		Second-order	*k*_2_ = 7.5 × 10^−2^ g mg^−1^ min^−1^	0.996

The situation with the adsorption of diazinon on both surfaces is not so clear-cut; even though *R*^2^ values for all the kinetic forms are ≥ 0.9, the plot for pseudo second-order behaviour has the *R*^2^ value closest to unity. Literature reports on the kinetic behaviour of both adsorbates show that both pesticides are adsorbed by second-order kinetics on a variety of surfaces. For example, the adsorption of diazinon on surfaces diverse as acid-activated bentonite^64^, surfactant-modified montmorillonites^65^, a magnetic composite of clay/graphene oxide/Fe_3_O_4_^66^, and NH_4_Cl-induced activated carbon^67^, all proceed as pseudo second-order processes. Similarly, the adsorption of dichlorvos on coconut fibre biochar^68^, on soil surfaces^69^ and on polyethyleneimine-modified fibres^70^ are all second-order processes. The data in Table 3 show that both diazinon and dichlorvos are adsorbed at comparable rates on CDA and starch, the ratio of the second-order rate constant for the adsorption of dichlorvos, $k_{2}^{dichl}$, and diazinon, $k_{2}^{diaz}$, i.e., $\frac{{k_{2}^{dichl} }}{{k_{2}^{diaz} }}$ being merely 0.8 and 1.6 for CDA and starch, respectively. Since the ratio $\frac{{K_{L}^{dichl} }}{{K_{L}^{diaz} }}$ for the adsorption of these pesticides on both surfaces is 1.3 (see Table 2), it then appears that these two surfaces, CDA and starch, do not differ substantially in their thermodynamic and kinetic responses towards these adsorbents.

### Kinetics of the desorption of the pesticides from CDA and starch surfaces into water

The release of the pesticides from CDA and starch surfaces into water was studied at pH 7.0 using the decanting method described by Cruz-Guzman et al.^29^ The experimental data obtained for the desorption of diazinon and dichlorvos from CDA and starch surfaces are assembled in Tables S8 and S9 (Supplementary Information), respectively. The kinetics data for desorption of the adsorbates from the surfaces were fitted to the linear forms of pseudo zero-, pseudo first-, and pseudo second-order behaviour, using Eqs. (8)-(10), as was the case for adsorption kinetics (vide supra).

**Figure 4:**
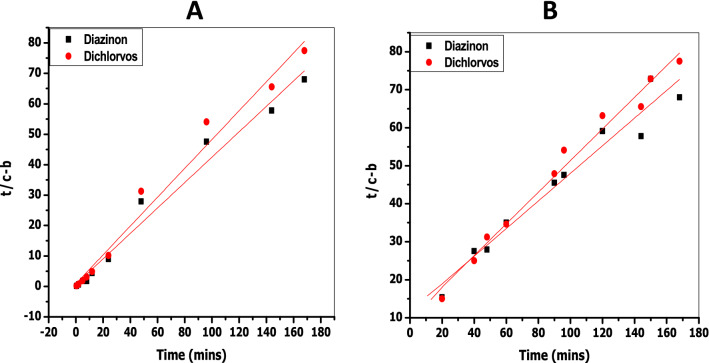
Second-order kinetic plots for the desorption of diazinon and dichlorvos from (**A**) CDA surface and (**B**) from starch surface into water at pH 7.0 and 27 °C.

The plots for zero-order and first-order desorption are shown in Figs. S5 and S6 (Supplementary Information), respectively, while those for second-order behaviour of both pesticides on CDA and starch surfaces are shown in Fig. 4. The rate constants and *R*^2^ values extracted from these plots are displayed in Table 5. It is clear from the *R*^2^ values in Table 5, obtained from the plots in Figures S5, S6 (Supplementary Information) and 4, that the desorption kinetic data give the best fit for the two pesticides when modelled according to pseudo second-order behaviour on both surfaces. The ratio $\frac{{k_{ - 2}^{dichl} }}{{k_{ - 2}^{dioz} }} = 2$ and 1 for CDA and starch, respectively, shows that dichlorvos desorbs from CDA surface twice as fast as diazinon, whilst both pesticides desorb from starch at similar rates. On the other hand, the ratio $\frac{{k_{ - 2}^{CDA} }}{{k_{ - 2}^{starch} }} = 0.5$ and 1 for diazinon and dichlorvos, respectively, shows that there is just a twofold difference in the desorption rates of both pesticides on going from CDA to starch. In other words, the second-order rate constants for the desorption of the two pesticides from the two surfaces are all of the same order of magnitude.

**Table 5:** Rate constants and *R*^2^ values obtained by modelling the desorption of diazinon and dichlorvos from the CDA and starch surfaces into water at pH 7.0 and 27 °C.

Surface	Pesticide	Order of reaction	Rate constant	*R*^2^ value
CDA	Diazinon	Zero-order	*k*_*-*0_ = 3.0 × 10^−3^ mg min^−1^	0.763
		First-order	*k*_*-*1_ = 1.0 × 10^−3^ min^−1^	0.760
		Second-order	*k*_*-*2_ = 1.1 × 10^−1^ g mg^−1^ min^−1^	0.973
	Dichlorvos	Zero-order	*k*_*-*0_ = 3.0 × 10^−3^ mg min^−1^	0.818
		First-order	*k*_*-*1_ = 1.0 × 10^−3^ min^−1^	0.851
		Second-order	*k*_*-*2_ = 2.3 × 10^−1^ g mg^−1^ min^−1^	0.998
Starch	Diazinon	Zero-order	*k*_*-*0_ = 4.0 × 10^−3^ mg min^−1^	0.711
		First-order	*k*_*-*1_ = 1.9 × 10^−3^ min^−1^	0.767
		Second-order	*k*_*-*2_ = 2.1 × 10^−1^ g mg^−1^ min^−1^	0.998
	Dichlorvos	Zero-order	*k*_*-*0_ = 3.0 × 10^−3^ mg min^−1^	0.638
		First-order	*k*_*-*1_ = 2.0 × 10^−3^ min^−1^	0.712
		Second-order	*k*_*-*2_ = 2.2 × 10^−1^ g mg^−1^ min^−1^	0.985

**Figure 5:**
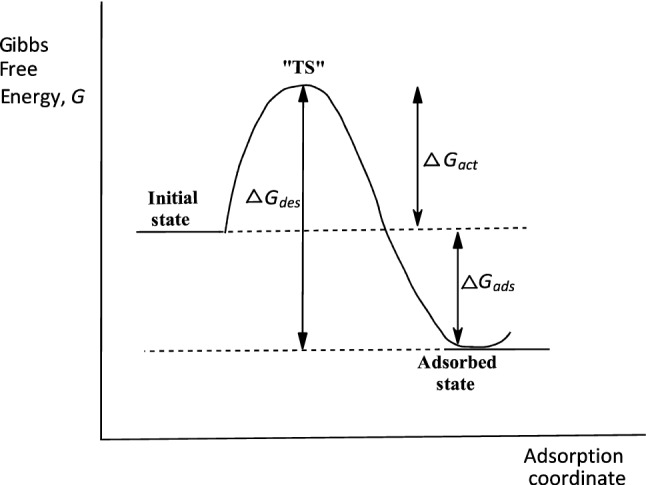
A two-dimensional Gibbs free energy diagram in which an adsorption process is treated as a chemical reaction, showing the energy barriers for adsorption and desorption with a hypothetical transition state “TS”. Desorption is seen as the microscopic reverse of adsorption. Adapted, with permission, from the copyright holder, M. A. Hubbe (see^74^).

Our kinetic data show that both the forward and reverse directions in the adsorption of the two pesticides follow second-order kinetics. The reverse process is therefore the microscopic reverse^71–73^ of the forward process, as illustrated in the free energy profile in Fig. 5, adapted from the paper by Hubbe et al.^74^ In Fig. 5, the free energy change for desorption, Δ*G*_*des*_, is a composite term which is related to the free energy change for adsorption, Δ*G*_*ads*_, according to Eq. (11), where Δ*G*_*act*_ is the free energy change of “activation,” which could be regarded as the energy required to prepare the vacant sites on the polymer for adsorption. The microscopic reverse of this process of “activation” as desorption takes place would entail the energy given out as the polymer surface returns to normality. These free energy terms are the energies required to overcome the barriers associated with adsorption, desorption, and “activation”. Consequently, the quantities, *k*_*ads*_* k*_*des*_ and *k*_*act*_, being the rate constants associated with overcoming the barriers to adsorption, desorption, and “activation,” are related to Δ*G*_*ads*_, Δ*G*_*des*_, and Δ*G*_*act*_, respectively, according to the generalized Eyring expression^71,72^ in Eq. (12), in which *k*_*i*_ = a rate constant, Δ*G*_*i*_ = free energy change associated with *k*_*i*_, *k* = the Boltzmann constant, *h* = the Planck constant, and *T* = absolute temperature. It is a fairly settled issue that adsorption and desorption are related by microscopic reversibility^75,76^. Fang et al.^77^, for example, have shown that the principle of microscopic reversibility is fulfilled by the rate constants for adsorption and desorption of proteins on cellulosic surfaces.

11 $$\Delta G_{des} = \Delta G_{ads} + \Delta G_{act}$$

12 $$k_{i} = \frac{kT}{h}e^{{ - \left( {\Delta G_{i} /RT} \right)}}$$

The second-order behaviour of the forward (adsorption) and reverse (desorption) processes, as observed in this study, could be interpreted mechanistically to mean that either the diffusion of the adsorbate from the bulk solution to the polymer surface or the transport of the particle into the interior of the polymers, or both, are important events kinetically in the adsorption process^78,79^. The scope of the data does not enable a choice of which of these two processes is rate-limiting or, in fact, whether both steps are partially rate-limiting. The important finding from the kinetics herein reported is the comparable behaviour of the two surfaces, CDA and starch, towards the adsorption and desorption of the two pesticides.

## Conclusions

The FTIR spectrum of CDA shows that there are functional groups and molecular fragments in this matrix which are also found in starch. The XRD spectrum informs that the matrix CDA is substantially crystalline. The adsorption of diazinon and dichlorvos, two organophosphorus pesticides widely used in tropical agriculture, on the two polymeric surfaces, CDA and starch, follow both the Langmuir and Freundlich adsorption models, with the Langmuir isotherm giving slightly better fits (*R*^2^ ≥ 0.90) than its Freundlich counterpart (*R*^2^ ≥ 0.81). The positive values of the Langmuir parameters *K*_*L*_ and *R*_*L*_ indicate that the adsorption of the pesticides on the two surfaces is favourable, while the range of the Δ*G*_*ads*_ values evaluated from the Langmuir *K*_*L*_ values, points to physisorption as the adsorption type. Data for the forward (adsorption) and reverse (desorption) processes are best modelled by second-order kinetics. This kinetic form in the forward and reverse directions accord with the principle of microscopic reversibility. Our isothermal and kinetics results show that CDA, a waste material that is readily available at no cost, yields adsorption and kinetic parameters which are of the same order of magnitude as those of starch. These results suggest that CDA is potentially viable for deployment as a matrix for the formulation of low-cost controlled pesticide release devices. The foregoing implies that CDA could serve the same purpose as, and therefore be a substitute for, starch in controlled release formulations of the two pesticides, as an example of sustainable and beneficial bioresource utilization. From this perspective, it would be important to follow up this study by preparing controlled release formulations of diazinon and dichlorvos with starch and CDA as matrices. A comparison of the in vitro behaviour of the resulting formulations will enable an assessment of the promise which cow dung holds as a low-cost substitute for starch in this emerging agrochemical technology for the promotion of sustainable agriculture. Such studies are under active consideration in our laboratories.

## Supplementary Information

Supplementary Information.

## Data Availability

All data generated or analysed during this study are included in this published article [and its supplementary information files].
