# Supplementary material for: Comparative adsorptive behaviour of cow dung ash and starch as potential eco-friendly matrices for controlled organophosphorus pesticides delivery
Source: Sci Rep. 2022 Jul 1;12:11169. doi: 10.1038/s41598-022-15292-6 (PMC9249786; doi:10.1038/s41598-022-15292-6)
Supplement: Supplementary file 1 — Supplementary Information. [file 41598_2022_15292_MOESM1_ESM.docx]

**COMPARATIVE ADSORPTIVE BEHAVIOUR OF COW DUNG ASH AND STARCH AS POTENTIAL ECO-FRIENDLY MATRICES FOR CONTROLLED ORGANOPHOSPHORUS PESTICIDES DELIVERY**

Chinyere Emmanuella Okafor^1,2^ & Ikenna Onyido^1^*

^1^Department of Pure and Industrial Chemistry, Nnamdi Azikiwe University, Awka, Nigeria

^1,2^Department of Science Education, Chukwuemeka Odumegwu Ojukwu University,

Uli, Anambra State, Nigeria

**SUPPLEMENTARY INFORMATION**

**Table 1.** Compositional concentrations of chemicals used in this study

_______________________________________________________

**Chemical** **Concentration**

_______________________________________________________

Diazinon 1000 $\mu$g/ml

Dichlorvos 1000 $\mu$g/ml

Potassium phthalate pH 4.0*

Potassium dihydrogen sulphate pH 7.0*

Sodium tetraborate decahydrate pH 9.0*

Ninhydrin 0.02 mol/ml

Sodium hydroxide 0.1 mol/ml

________________________________________________________

*These were supplied as buffer solutions of known pH. The pH of these

solutions were checked before use.

**Table S2.** Experimental data for the adsorption of diazinon on CDA surface at pHs 4.0, 7.0 and 9.0*

| pH 4 | ***C_o_* (mg/L)** | ***C_e_* (mg/L)** | | ***C_o_-C_e_*** | ***q*_e_ (mg/g)** | **1/*q*_e_** | | **1/*C_e_*** | | **Log *q_e_*** | **Log *C_e_*** | |  |
| --- | --- | --- | --- | --- | --- | --- | --- | --- | --- | --- | --- | --- | --- |
|  | 1.0  5.0  10.0  15.0  20.0  25.0 | 0.3587  2.0440  2.7202  2.9248  3.1853  4.4651 | | 0.6413  2.956  7.2798  12.0752  16.8147  20.5349 | 0.1283  0.5912  1.4560  2.4150  3.3629  4.1070 | 7.7942  1.6915  0.6868  0.4141  0.2974  0.2435 | | 2.7878  0.4892  0.3676  0.3419  0.3139  0.2240 | | -0.8918  -0.2282  0.1632  0.3829  0.5267  0.6135 | -0.4453  0.3105  0.4346  0.4661  0.5032  0.6498 | |  |
|  | | | | | | | | | | | |  |  |
| pH 7 | ***C_o_* (mg/L)** | ***C_e_* (mg/L)** | ***C_o_-C_e_*** | | ***q*_e_ (mg/g)** | **1/*q*_e_** | **1/*C_e_*** | | **Log *q_e_*** | | **Log *C_e_*** | | |
|  | 1.0  5.0  10.0  15.0  20.0  25.0 | 0.3835  1.8067  2.0629  3.0468  3.5688  4.9853 | 0.6165  3.1933  7.9371  11.9532  16.4312  20.0147 | | 0.1233  0.6387  1.5874  2.3906  3.2863  4.0029 | 8.1103  1.5657  0.6300  0.4183  0.3043  0.2498 | 2.6076  0.5535  0.4848  0.3282  0.2802  0.2006 | | -0.1990  -0.1947  0.2007  0.3785  0.5167  0.6024 | | -0.4162  0.2569  0.3145  0.4838  0.5525  0.6977 | | |

| pH 9 | ***C_o_* (mg/L)** | ***C_e_* (mg/L)** | ***C_o_-C_e_*** | ***q*_e_ (mg/g)** | **1/*q*_e_** | **1/*C_e_*** | **Log *q_e_*** | **Log *C_e_*** |
| --- | --- | --- | --- | --- | --- | --- | --- | --- |
|  | 1.0  5.0  10.0  15.0  20.0  25.0 | 0.4073  1.9501  2.7000  3.0349  3.7083  4.6755 | 0.5927  3.0499  7.3000  11.9651  16.2917  20.3245 | 0.1185  0.6100  1.4600  2.3930  3.2583  4.0649 | 8.4388  1.6393  0.6849  0.4179  0.3069  0.2460 | 2.4552  0.5128  0.3704  0.3295  0.2697  0.2139 | -0.9263  -0.2147  0.1644  0.3789  0.5130  0.6090 | -0.3901  0.2901  0.4314  0.4821  0.5692  0.6698 |

*The measured quantities were conducted in duplicates and are subject to experimental uncertainties of 5 – 7%.

**Table S3.** Experimental data for the adsorption of dichlorvos on CDA surface at pH 4.0, 7.0 and 9.0*

| pH 4 | ***C_o_* (mg/L)** | ***C_e_* (mg/L)** | ***C_o_-C_e_*** | ***q*_e_ (mg/g)** | **1/*q*_e_** | **1/*C_e_*** | **Log *q_e_*** | **Log *C_e_*** |
| --- | --- | --- | --- | --- | --- | --- | --- | --- |
|  | 1.0  5.0  10.0  15.0  20.0  25.0 | 0.5987  3.0423  7.0154  9.7231  11.0872  12.1423 | 0.4014  1.9577  2.9846  5.2769  8.9128  12.8577 | 0.0803  0.3915  0.5969  1.0554  1.7826  2.5715 | 12.4579  2.5540  1.6753  0.9475  0.5610  0.3889 | 1.6703  0.5108  0.1425  0.1029  0.0902  0.0824 | -1.0953  -0.4073  -0.2241  0.0234  0.2511  0.4102 | -0.2228  0.4832  0.8461  0.9878  1.0448  1.0843 |

| pH 7 | ***C_o_* (mg/L)** | ***C_e_* (mg/L)** | ***C_o_-C_e_*** | ***q*_e_ (mg/g)** | **1/*q*_e_** | **1/*C_e_*** | **Log *q_e_*** | **Log *C_e_*** |
| --- | --- | --- | --- | --- | --- | --- | --- | --- |
|  | 1.0  5.0  10.0  15.0  20.0  25.0 | 0.4638  1.8987  3.2167  3.6718  5.5308  6.4179 | 0.8526  3.1013  6.7833  11.3282  14.4692  18.5821 | 0.1190  0.6203  1.3567  2.2656  2.8938  3.7164 | 2.4765  0.5351  0.3109  0.2724  0.1808  0.1558 | 8.4034  1.6121  0.7371  0.4414  0.3456  0.2691 | -0.3938  0.2785  0.5074  0.5649  0.7428  0.8074 | -0.9245  -0.2074  0.1325  0.3552  0.4615  0.5701 |

| pH 9 | ***C_o_* (mg/L)** | ***C_e_* (mg/L)** | ***C_o_-C_e_*** | ***q*_e_ (mg/g)** | **1/*q*_e_** | **1/*C_e_*** | **Log *q_e_*** | **Log *C_e_*** |
| --- | --- | --- | --- | --- | --- | --- | --- | --- |
|  | 1.0  5.0  10.0  15.0  20.0  25.0 | 0.1379  1.7297  3.7423  5.7808  6.2051  7.2256 | 0.8603  3.2603  6.2577  9.2192  13.7949  17.7744 | 0.1721  0.6521  1.2515  1.8439  2.7549  3.5549 | 5.8122  1.5336  0.7990  0.5424  0.3625  0.2813 | 7.1582  0.5748  0.2672  0.1730  0.1612  0.1384 | -0.7642  -0.1857  0.0974  0.2657  0.4408  0.5508 | -0.8548  0.2405  0.5731  0.7620  0.7927  0.8689 |

*The measured quantities were conducted in duplicates and are subject to experimental uncertainties of 5 – 7%.

**Table S4**. Experimental data for the adsorption of diazinon on starch at pH 4.0, 7.0 and 9.0 and 27^o^C*

| pH 4 | **C_o_ (mg/L)** | **C_e_ (mg/L)** | **C_o_-C_e_** | **Q_e_ (mg/g)** | **1/Q_e_** | **1/C_e_** | **Log Q_e_** | **Log C_e_** |
| --- | --- | --- | --- | --- | --- | --- | --- | --- |
|  | 1.0  5.0  10.0  15.0  20.0  25.0 | 0.1647  0.2857  0.6679  0.7688  1.0743  1.9849 | 0.8353  4.7143  9.3321  14.2312  18.9257  23.0151 | 0.1671  0.9429  1.8664  2.8462  3.7851  4.8030 | 6.0716  3.5002  1.4972  1.3007  0.9308  0.5038 | 5.9844  1.0606  0.5358  0.3513  0.2642  0.2172 | -0.7833  -0.5441  -0.1753  -0.1142  0.03113  0.2977 | -0.7770  -0.5441  -0.1753  -0.1142  0.0311  0.2977 |

| pH 7 | C_o_ (mg/L) | C_e_ (mg/L) | C_o_-C_e_ | Q_e_ (mg/g) | 1/Q_e_ | 1/C_e_ | Log Q_e_ | Log C_e_ |
| --- | --- | --- | --- | --- | --- | --- | --- | --- |
|  | 1.0  5.0  10.0  15.0  20.0  25.0 | 0.2752  0.5459  0.9835  1.3193  1.5596  1.7385 | 0.7248  4.4541  9.0165  13.6807  18.4404  23.2615 | 0.1445  0.8908  1.8033  2.7361  3.6881  4.6523 | 3.6337  1.8318  1.0168  0.7580  0.6412  0.5752 | 6.8985  1.1226  0.5545  0.3655  0.2711  0.2149 | -0.8401  -0.0502  0.2561  0.4371  0.5668  0.6677 | 0.0588  0.1734  0.2561  0.3296  0.3959  0.4552 |

| pH 9 | C_o_ (mg/L) | C_e_ (mg/L) | C_o_-C_e_ | Q_e_ (mg/g) | 1/Q_e_ | 1/C_e_ | Log Q_e_ | Log C_e_ |
| --- | --- | --- | --- | --- | --- | --- | --- | --- |
|  | 1.0  5.0  10.0  15.0  20.0  25.0 | 0.3706  0.7431  1.0459  1.2844  1.7220  2.2569 | 0.6294  4.2569  8.9541  13.7156  18.2780  22.7461 | 0.1259  0.8514  1.7909  2.7431  3.6556  4.5486 | 2.6983  1.3457  0.9561  0.7786  0.5807  0.4431 | 7.9428  1.1745  0.5584  0.3646  0.2736  0.2198 | -0.4311  -0.1290  0.0195  0.1087  0.2360  0.3535 | -0.9000  -0.0699  0.2531  0.4382  0.5630  0.6579 |

*The measured quantities were conducted in duplicates and are subject to experimental uncertainties of 5 – 7%.

**Table S5**. Experimental data for the adsorption of dichlorvos on starch at pH 4.0, 7.0 and 9.0 and 27^o^C*

| pH 4 | **C_o_ (mg/L)** | **C_e_ (mg/L)** | **C_o_-C_e_** | **Q_e_ (mg/g)** | **1/Q_e_** | **1/C_e_** | **Log Q_e_** | **Log C_e_** |
| --- | --- | --- | --- | --- | --- | --- | --- | --- |
|  | 1.0  5.0  10.0  15.0  20.0  25.0 | 0.3654  1.9936  5.8269  7.3936  8.9782  10.0782 | 0.6346  3.0064  4.1731  7.6064  11.0218  14.9218 | 0.1269  0.6013  0.8346  1.5213  2.2044  2.9844 | 7.8790  1.6631  1.1982  0.6573  0.4537  0.3351 | 2.7367  0.5016  0.1716  0.1353  0.1114  0.0992 | -0.8965  -0.2209  -0.0785  0.1822  0.3433  0.4749 | -0.4372  0.2996  0.7654  0.8689  0.9532  1.0034 |

| pH 7 | C_o_ (mg/L) | C_e_ (mg/L) | C_o_-C_e_ | Q_e_ (mg/g) | 1/Q_e_ | 1/C_e_ | Log Q_e_ | Log C_e_ |
| --- | --- | --- | --- | --- | --- | --- | --- | --- |
|  | 1.0  5.0  10.0  15.0  20.0  25.0 | 0.1051  1.9051  2.6897  4.7205  5.7462  6.3526 | 0.8949  3.0949  7.3103  10.2795  14.2538  18.6474 | 0.1790  0.6190  1.4621  2.0559  2.8508  3.7295 | 9.5147  0.5128  0.3718  0.2118  0.1740  0.1574 | 5.5866  1.6155  0.6839  0.4864  0.3508  0.2681 | -0.9784  0.2901  0.4297  0.6740  0.7594  0.8030 | -0.47  -0.2083  0.1650  0.3130  0.4550  0.5717 |

| pH 9 | C_o_ (mg/L) | C_e_ (mg/L) | C_o_-C_e_ | Q_e_ (mg/g) | 1/Q_e_ | 1/C_e_ | Log Q_e_ | Log C_e_ |
| --- | --- | --- | --- | --- | --- | --- | --- | --- |
|  | 1.0  5.0  10.0  15.0  20.0  25.0 | 0.3244  0.6590  0.9192  1.1321  1.1590  1.6321 | 0.6756  4.3410  9.0808  13.8680  18.8410  23.3680 | 0.1351  0.8682  1.8162  2.7736  3.7682  4.6736 | 7.4004  1.1518  0.5506  0.3605  0.2654  0.2140 | 3.8261  1.5175  1.0879  0.8833  0.8628  0.6127 | -0.7314  0.0141  0.2799  0.4557  0.5798  0.6788 | -1.8277  -0.7951  -0.3239  -0.1408  -0.0013  0.0553 |

*The measured quantities were conducted in duplicates and are subject to experimental uncertainties of 5 – 7%.

**Table S6**. Data for the modelling of the adsorption kinetics of (a) diazinon and (b) dichlorvos on CDA surface according to zero-, first- and second-order behaviour at pH 7.0 and 27^0^C.

1. **Adsorbate = diazinon**

| **Time, *t* (mins)** | **(*c-b*)^a^ ln (*c/c-b*)^a^ *t/*(*c-b*)^a^** |
| --- | --- |
| 20  40  60  90  120  150 | 1.369 0.314 14.609  1.519 0.418 26.342  1.937 0.661 30.981  2.121 0.752 42.425  2.321 0.842 51.697  2.547 0.935 58.900 |

^a^See the text for the definition of these notations.

1. **Adsorbate = dichlorvos**

| **Time, *t* (mins)** | **( *c-b*)^a^ ln (*c/c-b*)^a^ *t/*(*c-b*)^a^** |
| --- | --- |
| 20  40  60  90  120  150 | 2.673 0.983 14.209  4.332 1.466 25.642  6.555 1.880 29.951  6.940 1.932 32.969  7.602 2.029 45.697  8.725 2.161 50.193 |

^a^See the text for the definition of these notations.

**Table S7**. Data for the modelling of the adsorption kinetics of (a) diazinon and (b) dichlorvos on starch surface according to zero-, first- and second-order behaviour at pH 7.0 and 27^0^C.

1. **Adsorbate = diazinon**

| **Time, *t* (mins)** | **( *c-b*)^a^ ln (*c/c-b*)^a^ *t/*(*c-b*)^a^** |
| --- | --- |
| 0.10  10  20  40  60  90  120  150 | 1.090 0.086 0.092  1.238 0.214 8.075  1.301 0.263 15.376  1.451 0.372 27.567  1.713 0.538 35.020  1.978 0.682 45.512  2.031 0.708 59.099  2.058 0.722 72.883 |

^a^See the text for the definition of these notations.

1. **Adsorbate = dichlorvos**

| **Time, *t* (mins)** | **( *c-b*)^a^ ln (*c/c-b*)^a^ *t/*(*c-b*)^a^** |
| --- | --- |
| 0.1  10  20  40  60  90  120  150 | 1.193 0.177 0.084  1.250 0.223 8.003  1.333 0.287 15.005  1.600 0.470 24.995  1.733 0.550 34.630  1.878 0.630 47.918  1.900 0.642 63.171  1.951 0.668 72.883 |

^a^See the text for the definition of these notations.

**Table S8**. Data for the desorption of diazinon and dichlorvos from CDA surface at 27^o^C and pH 7.0

1. **Desorbate = Diazinon**

| **Time (hrs.)** | **Abs C_e_** |
| --- | --- |
| 0.5  2.0  5.0  8.0  12.0  24.0  48.0  96.0  144.0  168.0  192.0 | 0.0254 0.0318  0.0272 0.0478  0.0293 0.0671  0.0300 0.0733  0.0312 0.0845  0.0322 0.094  0.0333 0.1045  0.0342 0.1121  0.0346 0.1163   - 1. 0.1191   0.3630 0.1317 |

1. **Desorbate = Dichlorvos**

| **Time (hrs)** | **Abs C_e_** |
| --- | --- |
| 0.5  2.0  5.0  8.0  12.0  24.0  48.0  96.0  144.0  168.0  192.0 | 0.1244 0.0055  0.1245 0.0073  0.1249 0.0116  0.1252 0.0156  0.1262 0.0284  0.1269 0.0369  0.1276 0.0455  0.1279 0.0503  0.1288 0.0616  0.1291 0.0659  0.1292 0.0671 |

**Table S9.** Data for the desorption of diazinon and dichlorvos from starch surface at 27^o^C and pH 7.0

1. **Desorbate = Diazinon**

| **Time (hrs.)** | **Abs C_e_** |
| --- | --- |
| 0.5  2.0  5.0  8.0  12.0  24.0  48.0  96.0  144.0  168.0  192.0 | 0.0292 0.0664  0.0309 0.0817  0.0344 0.1139  0.0365 0.1331  0.0392 0.1583  0.0402 0.1674  0.0414 0.1785  0.0432 0.1946  0.0452 0.2128  0.0458 0.2184   - 1. 0.2230 |

1. **Desorbate = Dichlorvos**

| **Time (hrs.)** | **Abs C_e_** |
| --- | --- |
| 0.5  2.0  5.0  8.0  12.0  24.0  48.0  96.0  144.0  168.0  192.0 | 0.1265 0.0332  0.1291 0.0658  0.1316 0.0982  0.1333 0.1201  0.1343 0.1332  0.1351 0.1433  0.1359 0.1519  0.1368 0.1635  0.1378 0.1772  0.1385 0.1864  0.1394 0.1967 |

A B

**Figure S1.** Calibration plots according to the Beer—Lambert Law for the absorbances of (A) to yield $\varepsilon$ = 1.73 x 10^4^ L mol^-1^cm^-1^and (B) the derivative of dichlorvos with ninhydrin to yield $\varepsilon$ diazinon = 3.34 x 10^3^ L mol^-1^cm^-1^.

**A B**

**C D**

**Figure S2**. Freundlich isotherms, obtained by application of equation (7) in the text, at 27^o^C and pH 4.0, 7.0 and 9.0 for the adsorption of (A) diazinon and (B) dichlorvos on CDA and for the adsorption of (C) diazinon and (D) dichlorvos on starch. The plots have the following *R^2^* values at the stated pHs: (A) 0.907 (pH 4), 0.959 (pH 7), 0.953 (pH 9); (B) 0.926 (pH 4), 0.984 (pH 7), 0.854 (pH 9); (C) 0.843 (pH 4), 0.975 (pH 7), 0.941 (pH 9); and (D) 0.987 (pH 4), 0.808 (pH 7), 0.993.

**A B**

**Figure S3.** Zero-order kinetic plots, according to equation (8) in the text, for the adsorption of (A) diazinon and dichlorvos on CDA and for the adsorption of (B) diazinon and dichlorvos on starch at pH 7.0 and 27^o^C. The following *R^2^* values were obtained for the plots: (A) 0.962 (diazinon), 0.888 (dichlorvos) on CDA; and (B) 0.925 (diazinon), 0.888 (dichlorvos) on starch.

**A B**

**Figure S4**. First-order kinetic plots, according to equation (9) in the text, for the adsorption of (A) diazinon and dichlorvos on CDA and for the adsorption of (B) diazinon and dichlorvos on starch at pH 7.0 and 27^o^C. The following *R^2^* values were obtained for the plots: (A) 0.928 (diazinon), 0.792 (dichlorvos) on CDA; and (B) 0.899 (diazinon), 0.855 (dichlorvos) on starch.

**A B**

**Figure S5**. Zero-order kinetic plots, according to equation (8) in the text, for the desorption of (A) diazinon and dichlorvos from CDA and for the desorption of (B) diazinon and dichlorvos from starch into water at pH 7.0 and 27^o^C. The following *R^2^* values were obtained for the plots: (A) 0.763 (diazinon), 0.818 (dichlorvos) on CDA; and (B) 0.711 (diazinon), 0.638 (dichlorvos) on starch.

**A B**

******Figure S6.** First-order kinetic plots, according to equation (9) in the text, for the desorption of (A) diazinon and dichlorvos from CDA and for the desorption of (B) diazinon and dichlorvos into water at pH 7.0 and 27^o^C. The following *R^2^* values were obtained for the plots: (A) 0.760 (diazinon), 0.851(dichlorvos) on CDA; and (B) 0.767 (diazinon), 0.712 (dichlorvos) on starch.
